# YWHAZ loss is associated with endometrial dysfunction in proliferative-phase endometriosis

**DOI:** 10.1093/reprod/xaag038

**Published:** 2026-04-06

**Authors:** Alice Santos da Silva, Md Saidur Rahman, Eunhee M Jeong, Shamsun Nahar, Steven L Young, Bruce A Lessey, Jae-Wook Jeong, Tae Hoon Kim

**Affiliations:** Department of Obstetrics, Gynecology and Women’s Health, University of Missouri School of Medicine, Columbia, MO, United States; Department of Obstetrics, Gynecology and Women’s Health, University of Missouri School of Medicine, Columbia, MO, United States; Department of Obstetrics, Gynecology and Women’s Health, University of Missouri School of Medicine, Columbia, MO, United States; Department of Obstetrics, Gynecology and Women’s Health, University of Missouri School of Medicine, Columbia, MO, United States; Department of Obstetrics, Gynecology and Women’s Health, Duke University, Durham, NC, United States; Department of Obstetrics and Gynecology, Atrium Health Wake Forest Baptist, Winston-Salem, NC, United States; Department of Obstetrics, Gynecology and Women’s Health, University of Missouri School of Medicine, Columbia, MO, United States; Department of Obstetrics, Gynecology and Women’s Health, University of Missouri School of Medicine, Columbia, MO, United States

**Keywords:** endometriosis, endometrium, infertility, RNA-sequencing, YWHAZ

## Abstract

Endometriosis is an estrogen-dependent inflammatory disorder frequently associated with infertility and characterized by progesterone resistance and impaired endometrial receptivity. While ectopic lesions define the disease, accumulating evidence indicates that molecular abnormalities within the eutopic endometrium substantially contribute to infertility. In this study, we performed transcriptomic analysis of proliferative-phase eutopic endometrium from women with and without endometriosis and identified 44 dysregulated genes, including 13 upregulated and 31 downregulated genes, in women with endometriosis compared with controls. Among the significantly altered genes, YWHAZ (14-3-3 zeta) emerged as a prominently downregulated gene. Reduced YWHAZ expression was further validated at the protein level in both epithelial and stromal compartments of the endometrium from women with endometriosis. To investigate its physiological regulation, we examined YWHAZ expression during early pregnancy in mice and observed a dynamic, stage-specific pattern during the peri-implantation period, with strong expression at gestational days 3.5 and 4.5 and robust expression at the primary decidual zone at gestational day 5.5. Notably, YWHAZ expression was nearly abolished in uteri from progesterone receptor knockout and uterine signal transducer and activator of transcription 3 conditional knockout (*Pgr^cre/+^Stat3^f/f^*) mice, indicating that YWHAZ is dependent on intact progesterone receptor and STAT3 signaling. Together, these findings identify YWHAZ as a hormonally and transcriptionally regulated endometrial factor that is disrupted in endometriosis and tightly linked to implantation and decidual progression. This study highlights YWHAZ as a potential molecular node connecting progesterone resistance and STAT3 dysregulation to defective endometrial function and infertility in endometriosis.

## Teaser

In brief

Endometriosis is a chronic disease that affects women’s fertility. This study identifies differentially expressed genes in the eutopic endometrium of women with endometriosis during the proliferative phase and highlights YWHAZ as a potential mediator of uterine function and endometriosis-associated infertility.

Endometriosis is a chronic, estrogen-dependent inflammatory disorder affecting ∼10% of reproductive-age women—nearly 190 million worldwide—and remains a major cause of pelvic pain and infertility (Bonavina & Taylor, 2022; Pasalic et al., 2023). Although endometriosis is defined by the presence of endometrial-like tissue outside the uterus, the eutopic endometrium itself exhibits profound molecular abnormalities that contribute to impaired fertility (Bonavina & Taylor, 2022). Progesterone resistance, estrogen dominance, and a persistent inflammatory milieu collectively disrupt endometrial receptivity and limit successful implantation (Coccia et al., 2022). Despite extensive clinical characterization, the molecular mechanisms that initiate and sustain these abnormalities remain poorly understood, hindering the development of diagnostic biomarkers and targeted therapies.

Because endometrial biology is tightly regulated by cyclical hormonal cues, transcriptomic changes during specific menstrual cycle phases can provide mechanistic insight into disease-associated alterations (Teh et al., 2023). The proliferative phase, characterized by active estrogen signaling and cell proliferation, provides an ideal window for investigating transcriptomic alterations associated with endometriosis (Apostolov et al., 2024). Under normal conditions, the endometrium undergoes morphological and molecular changes in response to fluctuating hormone levels, reflected in its transcriptomic regulation (Sigurgeirsson et al., 2017). However, in endometriosis, estrogen dominance and progesterone resistance disrupt these processes, leading to abnormalities in the eutopic proliferative endometrium compared with that of healthy women (Alotaibi, 2025). These alterations include dysregulation in the expression of genes involved in cell proliferation and migration, angiogenesis and signaling pathways, and increased inflammatory response, consequently leading to impaired endometrial receptivity (Adamczyk et al., 2022). Advances in high-throughput genomic technologies, including bulk RNA-sequencing, single-cell RNA-sequencing, and spatial transcriptomics, have facilitated deeper investigation of the eutopic endometrium in endometriosis (Fonseca et al., 2023; Zhang et al., 2025; Zheng et al., 2023). Previous transcriptomic studies have identified differential gene expression and dysregulated molecular pathways in the eutopic endometrium of women with endometriosis. Single-cell transcriptomic analyses further revealed alterations in cellular composition, including changes in the relative proportions of stromal, immune, and epithelial cell populations (Fonseca et al., 2023). Consistently, bulk RNA-sequencing studies have reported differentially expressed genes (DEGs) associated with extracellular matrix organization, angiogenesis, cell proliferation, and inflammatory responses (Zhao et al., 2017). Collectively, these transcriptomic approaches have provided critical insight into molecular pathways implicated in the pathogenesis of endometriosis (Chen et al., 2022).

YWHAZ (tyrosine 3-monooxygenase/tryptophan 5-monooxy­-genase activation protein, zeta polypeptide) encodes 14-3-3 zeta, a member of the 14-3-3 family of proteins, a highly conserved family of phospho-serine/threonine-binding regulatory proteins that act as adaptor molecules, modulating the stability, localization, and activity of numerous target proteins involved in cell cycle control, apoptosis, metabolism, differentiation, adhesion, and motility (Barrera et al., 2025; Neal & Yu, 2010). Many of these processes are disrupted in endometriosis, which is characterized by aberrant cell proliferation, impaired apoptosis, altered migration, and pronounced inflammatory signaling (Mariadas et al., 2025). Given the complexity of endometrial remodeling and its reliance on coordinated signaling networks, disruption of YWHAZ expression may have substantial consequences for uterine function.

In endometriosis, progesterone resistance not only impairs the hormonal balance but also influences the expression and activity of signaling molecules and transcriptional regulators (Patel et al., 2017). In particular, the signal transducer and activator of transcription 3 (STAT3), a transcription factor activated by several cytokines, has been identified as a key mediator in the disease. Aberrant STAT3 activation contributes to inflammation and defective decidualization, both of which are linked to progesterone resistance (Kim et al., 2015). Considering the interplay between progesterone resistance and STAT3 dysregulation in endometriosis, the regulation of YWHAZ by these pathways emerges as a potential target to better understand molecular mechanisms underlying endometriosis.

In this study, we examined transcriptomic profiles of eutopic endometrium from women with and without endometriosis during the proliferative phase and identified YWHAZ as a significantly downregulated gene in affected individuals. We validated YWHAZ protein expression in human endometrial tissues, characterized its dynamic regulation during early pregnancy in mice, and investigated its dependence on progesterone signaling and STAT3 activity using progesterone receptor knockout (PRKO) and *Stat3* conditional knockout (*Pgr^cre/+^Stat3^f/f^*) models. These findings highlight YWHAZ as a potential regulator of endometrial differentiation and decidualization, offering new insight into molecular pathways that may contribute to endometriosis-associated infertility.

## Materials and methods

### Ethics approval

All human uterine tissues used was approved by the Institutional Review Boards of Atrium Health Wake Forest Baptist (Winston-Salem, NC, USA) under IRB00057549. Written informed consent was obtained from all human subjects prior to tissue acquisition.

All animal experiments were approved by the University of Missouri Animal Care and Use Committee (protocol number 65323). Mice were housed and bred in a designated animal care facility at the University of Missouri with controlled humidity (30%–70%) and temperature (68–79°F) conditions and a 12 hr light/dark cycle.

### Human endometrial sample collection

Eutopic endometrium was obtained by endometrial biopsy from women between the ages of 18 and 45 years, including four with endometriosis and four without the disease, during the proliferative phase. The presence or absence of disease was confirmed in all endometriosis cases, and controls included women who were laparoscopically negative for disease. Use of an intrauterine device (IUD) or hormonal therapies in the 3 months preceding surgery was exclusionary. Histological dating of endometrial samples was performed by a certified pathologist.

### RNA isolation

Eutopic endometrial biopsies from control (*n* = 4) and endometriosis (*n* = 4) groups were used for RNA-sequencing analysis. Total RNA was isolated from each sample using the RNeasy Total RNA Isolation kit (Qiagen; Aarhus, Denmark) following the manufacturer’s instructions. RNA purity and concentration were analyzed by the Genomic Technology Core at the University of Missouri to confirm sample concentration and purity (50–200 ng/µl, RNA integrity number (RIN) > 8.0) before RNA-sequencing.

### RNA-sequencing analysis

For RNA-sequencing, libraries were prepared from 500 ng of total RNA using the KAPA mRNA Hyperprep kit (Kapa Biosystems; Wilmington, MA, USA). RNA was sheared to 300–400 bp. Prior to PCR amplification, cDNA fragments were ligated to Integrated DNA Technologies (IDT) for Illumina TruSeq UD Indexed adapters (Illumina Inc; San Diego, CA, USA). The quality and quantity of the finished libraries were assessed using a combination of Agilent DNA high-sensitivity chip (Agilent Technologies, Inc.; Santa Clara, CA, USA) and QuantiFluor^®^ dsDNA system (Promega Corp.; Madison, WI, USA). Individually indexed libraries were pooled, and 50 bp paired-end sequencing was performed on an Illumina NovaSeq6000 sequencer using an S2, 100 bp sequencing kit (Illumina Inc.; San Diego, CA, USA) to an average depth of 50 million reads per sample. Base calling was done by Illumina RTA3, and the output of NextSeq Control Software (NCS) was demultiplexed and converted into FastQ format with Illumina Bcl2fastq software (version 1.9.0).

The raw reads were initially trimmed and filtered by removing low-quality reads (fragments that scored < 20) and aligned to the hg38 human genome reference and assembled using STAR (version 2.7.11b) (Dobin et al., 2013). Expression values of RNA-sequencing were expressed as counts. DEGs were identified by the “exact negative binomial test” in the EdgeR software package (Robinson et al., 2010). The DEGs between control and endometriosis groups were set as fold-change >2 (upregulated genes) or <−2 (downregulated genes) and false discovery rate < 0.05. DEG expression values were normalized, scaled, and clustered to generate a heatmap using the ComplexHeatmap package (Gu et al., 2016).

### Availability of data and material

The raw and processed RNA‑sequencing data generated in this study have been deposited in the NCBI Gene Expression Omnibus (GEO) under accession number GSE315857, which is a dataset derived from GSE272606. The data that support the findings of this study are available from the article and supplementary information files (see online supplementary material).

### Animals and tissue collection

For the early pregnancy study, wild-type C57BL/6 female mice at 8 weeks of age were mated with wild-type C57BL/6 fertile male mice, and uterine samples from pregnant mice were obtained on different gestational days (GDs). The morning of vaginal plug is designated as GD 0.5. Uterine horns were collected at GDs 0.5, 2.5, 3.5, 4.5, 5.5, and 7.5 (*n* = 5 per time-point). Additionally, 8-week old litter-mate control, PRKO, and *Pgr^cre/+^Stat3^f/f^* female mice were mated with wild-type C57BL/6 fertile male mice and uterine horn samples were collected at GD 3.5 (*n* = 5 per group) (Jeong et al., 2005; Kim et al., 2015; Lee et al., 2013; Teasley et al., 2018). Uterine horns were fixed in 4% paraformaldehyde in 1× phosphate-buffered saline (PBS), dehydrated through ethanol washes, and embedded in paraffin blocks for immunostaining.

### Immunohistochemistry

Paraffin-embedded human endometrial samples from women with and without endometriosis (*n* = 10 per group) were sectioned at 5 µm thickness, and immunohistochemistry staining and analysis were performed as previously described (Kim et al., 2015; Rahman et al., 2025b). Briefly, the sections were deparaffinized in xylene and rehydrated through a graded ethanol series. After antigen blocking, the sections were incubated with 10% normal goat serum (Vector Laboratories; Newark, CA, USA) in PBS (pH 7.5) and subsequently incubated with primary antibody diluted in 10% normal goat serum in PBS overnight at 4 °C (anti-14-3-3 zeta, 1:500; CS7413, Cell Signaling Technology; Danvers, MA, USA). On the following day, the sections were washed in PBS and incubated with a secondary antibody for 1 hr at room temperature. Subsequently, they were incubated with horseradish peroxidase (Vector Laboratories) for 45 min. After PBS washes, immunoreactivity was visualized using the Vectastain Elite DAB kit (Vector Laboratories). Slides were counterstained with hematoxylin and saturated lithium carbonate. A semi-quantitative H-score system (0–300) was used to compare staining intensities (Kim et al., 2025). Quantification of H-scores was performed using Visiopharm image analysis software (Visiopharm, Hørsholm, Denmark). For the early pregnancy, PRKO, and *Pgr^cre/+^Stat3^f/f^* samples, paraffin blocks were sectioned at 5 μm thickness, and immunohistochemistry analyses were performed in the same way as for human tissue sections.

### Statistical analysis

For statistical analysis, Student’s t-test with unequal variance was used to compare data with only two groups. For data containing more than two groups, one-way ANOVA (analysis of variance) followed by Tukey’s post hoc test was used for multiple comparisons. All data are presented as mean ± SEM. The statistical significance was considered when *p *< 0.05. All statistical analyses were performed using the Prism version 10.2.0 package from GraphPad (GraphPad Prism; San Diego, CA, USA).

## Results

### Transcriptomic analysis revealed differential gene expression in the eutopic endometrium from women with endometriosis during the proliferative phase

**Figure 1:**
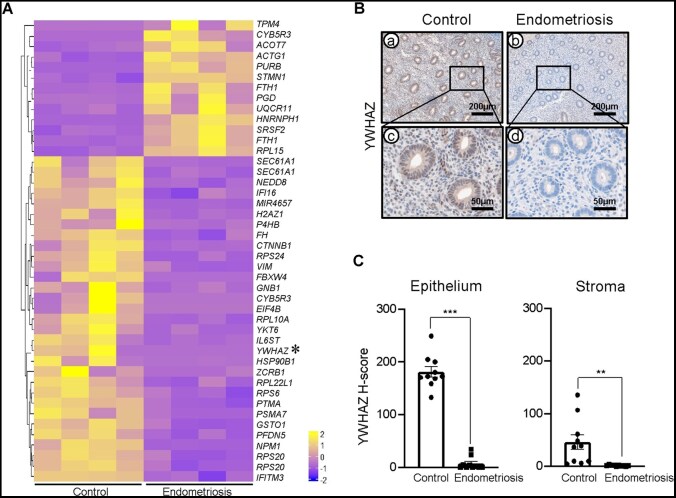
Transcriptomic analysis revealed differential gene expression in the eutopic endometrium from women with endometriosis during the proliferative phase. (A) Heatmap showing 44 differentially expressed genes in endometrial tissue of women with endometriosis (*n* = 4) compared to women without endometriosis (*n* = 4) during the proliferative phase (fold-change > ± 2.00; false discovery rate < 0.05). Upregulated and downregulated genes are color coded as yellow and blue, respectively. (B) Representative image of YWHAZ expression in human endometrial tissue. (C) H-score of YWHAZ expression in the endometriosis group (*n* = 10) compared to controls (*n* = 10). Results are presented as mean ± SEM; **p < 0.01, ***p < 0.001.

To identify differentially regulated genes in endometriosis, we performed RNA-sequencing analysis on eutopic endometrial tissue collected during the proliferative phase from women with and without endometriosis (*n* = 4 per group). A total of 44 DEGs were identified between control and endometriosis groups using a threshold value of fold-change > ± 2 and false discovery rate < 0.05. Among these, 13 genes were upregulated and 31 genes were downregulated in the endometriosis group. The expression profiles of these DEGs were visualized in a clustered heatmap, revealing a distinct gene expression pattern between the groups (Figure 1A).

Among the downregulated genes, YWHAZ was notable for its robust decrease in expression. To validate this result at the protein level, we performed immunohistochemistry on proliferative-phase eutopic endometrial tissue. A section processed without the primary antibody was used as the negative control for the human endometrium (supplementary Figure S1A, see online supplementary material). Consistent with the transcriptomic findings, YWHAZ immunoreactivity was markedly reduced in both stromal and epithelial compartments of endometriosis samples. Quantitative analysis revealed significantly lower YWHAZ staining intensity in the endometriosis group compared with controls (stroma: 46.09 ± 14.12 versus 1.79 ± 0.38, *p *< 0.01; and epithelium: 181.26 ± 9.91 versus 7.16 ± 3.83, *p *< 0.001) (Figure 1B and C). Together, these findings identify YWHAZ as a significantly downregulated gene in the eutopic endometrium of women with endometriosis during the proliferative phase.

### Dynamic YWHAZ expression pattern across early pregnancy

**Figure 2:**
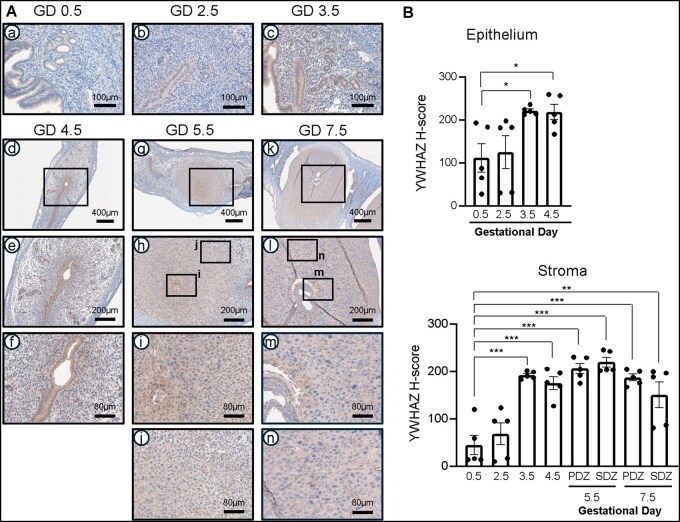
Dynamic YWHAZ expression pattern across early pregnancy. (A) Representative image of YWHAZ expression in uterine tissue from gestational day (GD) 0.5 to GD 7.5 (*n* = 5 per time point), showing the epithelial and stromal compartments, with the primary decidual zone (PDZ) and secondary decidual zone (SDZ) visible at GD5.5 and GD7.5. (B) H-score of YWHAZ expression during early pregnancy. Results are presented as mean ± SEM; *p < 0.05, **p < 0.01, ***p < 0.001.

To characterize the temporal expression pattern of YWHAZ during early pregnancy, we performed immunohistochemistry on uterine horns from wild-type C57BL/6 female mice collected from GD 0.5 to GD 7.5 (Figure 2; *n* = 5 per time point). YWHAZ expression was detected in the epithelium at GD 0.5 (111.81 ± 32.85) and GD 2.5 (124.94 ± 38.49). YWHAZ expression increased markedly in the uterine epithelium at GD 3.5 (221.66 ± 4.11; *p *= 0.010) and GD 4.5 (218.61 ± 17.80; *p *= 0.021) compared with GD 0.5. Stromal YWHAZ expression was lower at GD 0.5 (44.97 ± 20.71) and GD 2.5 (68.54 ± 23.07). Interestingly, a significant increase in YWHAZ expression was observed at GD 3.5 (192.57 ± 3.54, *p *< 0.001), GD 4.5 (175.24 ± 13.66; *p *< 0.001) in the primary decidual zone (206.67 ± 10.62; *p *< 0.001), GD5.5 in the secondary decidual zone (219.7 ± 10.00; *p *< 0.001), and in both the primary decidual zone and secondary decidual zone (187.6 ± 7.06 and 150.8 ± 27.19; *p *< 0.001 and *p *= 0.02, respectively) at GD 7.5 compared to GD 0.5. Together, these results suggest that YWHAZ has an important role during early implantation and pregnancy progression.

### Loss of YWHAZ expression in PGR knockout mice

**Figure 3:**
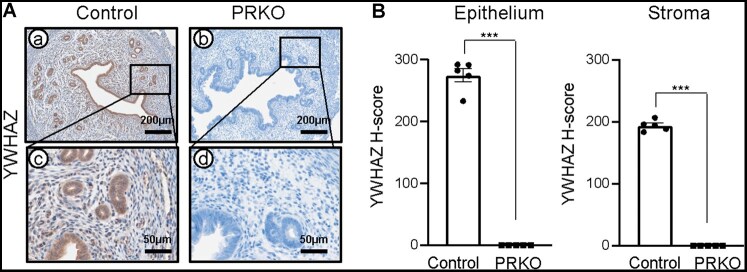
Loss of YWHAZ expression in progesterone receptor knockout (PRKO) mice. (A) Representative image of YWHAZ expression in the uterine tissue of control and PRKO mice. (B) H-score of YWHAZ expression in the uterine epithelium and stroma of control and PRKO mice (*n* = 5). Results are presented as mean ± SEM; ***p < 0.001.

Progesterone resistance refers to the failure of the endometrium to respond to progesterone. Many studies indicate that disrupted progesterone signaling and features of progesterone resistance are common in endometriosis (Zhang & Wang, 2023). To investigate whether YWHAZ contributes to the regulation of progesterone receptor (PGR) signaling, we examined YWHAZ expression in uteri from wild-type C57BL/6 (control) and PRKO female mice at GD 3.5. PRKO mice were validated by immunohistochemistry detection of PGR in the uterine tissue at GD3.5 (supplementary Figure S1B). Immunohistochemistry revealed strong YWHAZ expression in the uteri of controls whereas YWHAZ expression was not detected in the uteri of PRKO mice (epithelium 275.15 ± 10.88 versus 0.00 ± 0.00, *p *< 0.01; and stroma 194.02 ± 3.96 versus 0.00 ± 0.00, *p *< 0.001) (Figure 3). These findings indicate that YWHAZ expression is dependent on PGR, suggesting that YWHAZ acts downstream of PGR in regulating progesterone signaling.

### Loss of YWHAZ expression in Stat3 knockout mice

**Figure 4:**
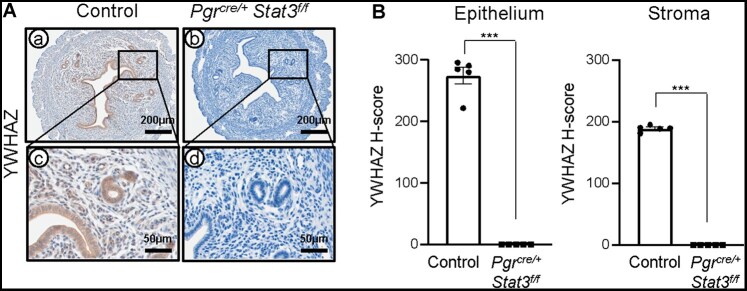
Loss of YWHAZ expression in signal transducer and activator of transcription 3 (*Stat3)* knockout mice. (A) Representative image of YWHAZ expression in the uterine tissue of control and *Pgr^cre/+^Stat3^f/f^* mice. (B) H-score of YWHAZ expression in uterine epithelium and stroma of control and *Pgr^cre/+^Stat3^f/f^* mice (*n* = 5). Results are presented as mean ± SEM; ***p < 0.001.

Our previous study demonstrated that STAT3 binds to the progesterone receptor A (PR-A) isoform, which is critical for uterine development and function, whereas no interaction occurs with PR-B (Lee et al., 2013). In addition, STAT3 regulates inflammatory and immune related genes, which are key contributors to the pathophysiology of endometriosis (Rahman et al., 2025a). In order to investigate the relationship between STAT3 and YWHAZ, we examined YWHAZ expression in the uteri of control and *Pgr^cre/+^Stat3^f/f^* mice at GD 3.5 using immunohistochemistry. Loss of STAT3 in the uteri of *Pgr^cre/+^Stat3^f/f^* mice at GD 3.5 was confirmed by immunohistochemistry (supplementary Figure S1C). In control mice, YWHAZ was strongly expressed in both stromal and epithelial compartments, whereas *Stat3* knockout uteri displayed a striking absence of YWHAZ expression (epithelium 274.99 ± 13.49 versus 0.00 ± 0.00; and stroma 189.27 ± 2.18 versus 0.00 ± 0.00, *p *< 0.001 respectively) (Figure 4). These results indicate that STAT3 activity is essential for maintaining YWHAZ levels during the peri-implantation period, suggesting that YWHAZ functions within the STAT3 pathway to regulate progesterone signaling.

## Discussion

In this study, we identified YWHAZ as one of the most markedly reduced genes in RNA-sequencing analysis of proliferative-phase eutopic endometrium, which revealed a distinct transcriptional profile in women with endometriosis compared with controls. Follow-up immunohistochemistry confirmed that YWHAZ protein expression was markedly decreased in both stromal and epithelial compartments of endometriosis samples, consistent with transcript-level changes. Together, these results highlight YWHAZ as a robustly downregulated gene in the eutopic endometrium of women with endometriosis, suggesting its potential relevance to disease-associated molecular alterations.

Moreover, we identified YWHAZ as a previously unrecognized gene of uterine function with potential relevance to endometrial receptivity and endometriosis-associated infertility. By integrating transcriptomic analysis of human eutopic endometrium with mechanistic interrogation in genetically engineered models, we demonstrated that YWHAZ expression is significantly reduced in the eutopic endometrium of women with endometriosis and is dynamically regulated during implantation and decidualization stages of early pregnancy in mice. Importantly, we show that PGR and STAT3 are required for uterine YWHAZ expression, positioning YWHAZ at the intersection of hormonal and cytokine-responsive transcriptional pathways critical for implantation.

Our transcriptomic analysis of proliferative-phase eutopic endometrium provides important context for understanding early molecular perturbations associated with endometriosis. The proliferative phase is dominated by estrogen-driven epithelial and stromal proliferation and precedes the establishment of progesterone responsiveness required for uterine receptivity. By focusing on this phase, our analysis captures disease-associated transcriptional alterations that are present before the window of implantation, thereby minimizing confounding effects of decidualization and highlighting intrinsic abnormalities of the eutopic endometrium. The identification of a discrete set of DEGs, including the marked downregulation of YWHAZ, suggests that endometriosis is associated with early disruptions in signaling pathways that may compromise subsequent progesterone-mediated differentiation and implantation competence. Importantly, the reduced expression of YWHAZ during the proliferative phase implies that defects in adaptor-mediated signaling networks may be established prior to the receptive window, potentially predisposing the endometrium to progesterone resistance and impaired decidual responses. These findings align with emerging transcriptomic and single-cell studies indicating that molecular abnormalities in the eutopic endometrium of women with endometriosis precede implantation and contribute to infertility independent of ectopic lesions.

YWHAZ encodes 14-3-3 zeta, a multifunctional adaptor protein that regulates the stability, subcellular localization, and activity of a broad array of signaling proteins and transcription factors (Neal & Yu, 2010). Through these interactions, 14-3-3 zeta integrates diverse cellular processes, including proliferation, differentiation, survival, and inflammatory signaling, that are tightly regulated in the normal endometrium and frequently disrupted in endometriosis. Thus, reduced YWHAZ expression in the eutopic endometrium of women with endometriosis may reflect impaired coordination of signaling networks necessary for establishing a receptive endometrial environment, thereby contributing to endometriosis-associated infertility.

To define the temporal regulation of YWHAZ during early pregnancy, we examined its expression across key stages of implantation and decidualization in the mouse uterus. Early pregnancy is characterized by tightly orchestrated hormonal and cellular transitions: estrogen-driven epithelial proliferation during the pre-receptive phase (GD 0.5–2.5), followed by progesterone-mediated stromal proliferation and differentiation coincident with uterine receptivity at GD 3.5. Blastocyst attachment occurs at GD 4.5, after which stromal cells undergo extensive proliferation and differentiation to form the primary and secondary decidual zones by GD 5.5–7.5. Our findings demonstrate that YWHAZ expression is low during the early stage but markedly induced in both epithelial and stromal compartments during the window of receptivity and throughout implantation progression. This dynamic, stage-specific pattern suggests that YWHAZ may contribute to the cellular remodeling and signaling integration required for successful implantation and decidualization. However, while our data establish a strong association between YWHAZ expression and these developmental events, direct functional studies will be required to define its precise mechanistic role. Our results further highlight the importance of hormonal and signaling crosstalk in regulating YWHAZ expression. Previous work has shown that progesterone induces another 14-3-3 isoform, 14-3-3τ, in uterine tissue, indicating that this protein family is responsive to progesterone signaling (Ito et al., 2012). Endometriosis patients who failed to improve with progestin-based treatments exhibited markedly reduced PGR expression compared with those who responded, indicating that PGR levels are closely associated with treatment responsiveness (Marquardt et al., 2019; Zhang & Wang, 2023). We observed a complete loss of YWHAZ expression in the uteri of PRKO mice, demonstrating that YWHAZ expression is strictly dependent on functional PGR signaling during the peri-implantation period and suggesting its potential as a therapeutic target to overcome progesterone resistance. Therefore, future studies should directly investigate the relationship between YWHAZ and PGR in implantation, decidualization, and endometriosis, as well as identify downstream molecular targets regulated by the PGR/YWHAZ axis in specific uterine cell types. These findings are particularly relevant in the context of endometriosis, where progesterone resistance is a defining feature of the eutopic endometrium.

In addition to progesterone signaling, STAT3 emerged as an essential regulator of YWHAZ expression. STAT3 is a key mediator of cytokine and growth factor signaling and has been implicated in uterine receptivity, decidualization, and the inflammatory milieu characteristic of endometriosis. Previous studies have shown that STAT3 can directly interact with YWHAZ through phosphorylation-dependent mechanisms, influencing transcriptional and signaling outputs (Han et al., 2015). Our results showing that YWHAZ expression is significantly reduced in uterine tissue of *Pgr^cre/+^Stat3^f/f^* mice provide in vivo evidence that STAT3 is required to maintain YWHAZ expression during early pregnancy. Together, these findings support a model in which progesterone receptor signaling and STAT3 converge to regulate YWHAZ expression, thereby integrating hormonal and inflammatory cues essential for normal uterine function.

Although this study provides compelling evidence that YWHAZ is hormonally and transcriptionally regulated in the uterus and disrupted in endometriosis, several limitations should be acknowledged. We did not directly assess the functional consequences of YWHAZ loss on implantation, decidualization, or fertility outcomes, nor did we define its downstream molecular targets in uterine cell types. Additionally, we did not evaluate whether YWHAZ functions within the STAT3 pathway to regulate progesterone signaling as a downstream effector of PGR in women with endometriosis. Future studies employing uterine cell-specific loss- and gain-of-function approaches, as well as mechanistic interrogation of YWHAZ-interacting proteins, will be critical to establish its causal role in endometrial dysfunction.

In summary, we identify YWHAZ as a downregulated gene in the eutopic endometrium of women with endometriosis and demonstrate that it exhibits dynamic, stage-specific expression during implantation and decidualization in the mouse uterus. The complete loss of YWHAZ expression in PGR and *Stat3* knockout models indicates that its expression is tightly regulated by progesterone receptor and STAT3. Collectively, these findings identify YWHAZ as a hormonally and transcriptionally regulated endometrial gene with potential relevance to uterine receptivity and endometriosis-associated infertility. Elucidating the functional role of YWHAZ may uncover new molecular mechanisms underlying progesterone resistance and provide novel therapeutic opportunities for reproductive disorders.

## Supplementary Material

xaag038_Supplementary_Data

## References

[xaag038-B1] Adamczyk M. , Wender-Ozegowska E., & Kedzia M. (2022). Epigenetic Factors in Eutopic Endometrium in Women with Endometriosis and Infertility. *International Journal of Molecular Sciences*, 23, 3804. 10.3390/ijms23073804 35409163 PMC8998720

[xaag038-B2] Alotaibi F. T. (2025). Pathophysiology of Endometriosis: Insights from Immunohistochemical Analysis of Ectopic and Eutopic Tissues. *International Journal of Molecular Sciences*, 26, 5998. 10.3390/ijms26135998 40649777 PMC12249977

[xaag038-B3] Apostolov A. , Naydenov M., Kalinina A., Nikolova M., Saare M., Aleksejeva E., Milova N., Milov A., Salumets A., Baev V., & Yahubyan G. (2024). Endometrial Proliferative Phase-Centered View of Transcriptome Dynamics across the Menstrual Cycle. *International Journal of Molecular Sciences*, 25, 5320. 10.3390/ijms25105320 38791358 PMC11121472

[xaag038-B4] Barrera E. E. , Skrabana R., Bustos D. M. (2025). Deciphering opening mechanisms of 14-3-3 proteins. Protein Science: a Publication of the Protein Society, 34, e70108. 10.1002/pro.70108 40130781 PMC11934215

[xaag038-B5] Bonavina G. , Taylor H. S. (2022). Endometriosis-associated infertility: From pathophysiology to tailored treatment. Front Endocrinol (Lausanne), 13, 1020827. 10.3389/fendo.2022.1020827 36387918 PMC9643365

[xaag038-B6] Chen C.-C. , Chou Y.-C., Hsu C.-Y., Tsai E.-M., & Er T.-K. (2022). Transcriptome Profiling of Eutopic and Ectopic Endometrial Stromal Cells in Women with Endometriosis Based on High-Throughput Sequencing. *Biomedicines*, 10, 2432. 10.3390/biomedicines10102432 36289693 PMC9598494

[xaag038-B7] Coccia M. E. , Nardone L., & Rizzello F. (2022). Endometriosis and Infertility: A Long-Life Approach to Preserve Reproductive Integrity. *International Journal of Environmental Research and Public Health*, 19, 6162. 10.3390/ijerph19106162 35627698 PMC9141878

[xaag038-B8] Dobin A. , Davis C. A., Schlesinger F., Drenkow J., Zaleski C., Jha S., Batut P., Chaisson M., Gingeras T. R. (2013). STAR: ultrafast universal RNA-seq aligner. Bioinformatics (Oxford, England), 29, 15–21. 10.1093/bioinformatics/bts635 23104886 PMC3530905

[xaag038-B9] Fonseca M. A. S. , Haro M., Wright K. N., Lin X., Abbasi F., Sun J., Hernandez L., Orr N. L., Hong J., Choi-Kuaea Y., Maluf H. M., Balzer B. L., Fishburn A., Hickey R., Cass I., Goodridge H. S., Truong M., Wang Y., Pisarska M. D., … Lawrenson K. (2023). Single-cell transcriptomic analysis of endometriosis. Nature Genetics, 55, 255–267. 10.1038/s41588-022-01254-1 36624343 PMC10950360

[xaag038-B10] Gu Z. , Eils R., Schlesner M. (2016). Complex heatmaps reveal patterns and correlations in multidimensional genomic data. Bioinformatics (Oxford, England), 32, 2847–2849. 10.1093/bioinformatics/btw313 27207943

[xaag038-B11] Han X. , Han Y., Jiao H., Jie Y. (2015). 14-3-3zeta regulates immune response through Stat3 signaling in oral squamous cell carcinoma. Molecules and Cells, 38, 112–121. 10.14348/molcells.2015.02101 25556369 PMC4332029

[xaag038-B12] Ito M. , Urano T., Hiroi H., Momoeda M., Saito M., Hosokawa Y., Tsutsumi R., Zenri F., Koizumi M., Nakae H., Horie-Inoue K., Fujii T., Yano T., Kozuma S., Inoue S., Taketani Y. (2012). The progesterone-responsive gene 14-3-3tau enhances the transcriptional activity of progesterone receptor in uterine cells. Journal of Molecular Endocrinology, 49, 193–202. 10.1530/JME-12-0112 22967481

[xaag038-B13] Jeong J. W. , Lee K. Y., Kwak I., White L. D., Hilsenbeck S. G., Lydon J. P., DeMayo F. J. (2005). Identification of murine uterine genes regulated in a ligand-dependent manner by the progesterone receptor. Endocrinology, 146, 3490–3505. 10.1210/en.2005-0016 15845616

[xaag038-B14] Kim B. G. , Yoo J. Y., Kim T. H., Shin J. H., Langenheim J. F., Ferguson S. D., Fazleabas A. T., Young S. L., Lessey B. A., Jeong J. W. (2015). Aberrant activation of signal transducer and activator of transcription-3 (STAT3) signaling in endometriosis. Human Reproduction (Oxford, England), 30, 1069–1078. 10.1093/humrep/dev050 25750101 PMC4400199

[xaag038-B15] Kim K. C. , Hull A., Johannsen E., Hunter M. I., Kim T. H., & Jeong J. W. (2025). ob/ob obese mice promote tumorigenesis of endometrial cancer associated with Pten deficiency. *Endocrine-Related Cancer*, 32, e240228. 10.1530/ERC-24-0228 40106332 PMC11957430

[xaag038-B16] Lee J. H. , Kim T. H., Oh S. J., Yoo J. Y., Akira S., Ku B. J., Lydon J. P., Jeong J. W. (2013). Signal transducer and activator of transcription-3 (Stat3) plays a critical role in implantation via progesterone receptor in uterus. FASEB Journal, 27, 2553–2563. 10.1096/fj.12-225664 23531596 PMC3688751

[xaag038-B17] Mariadas H. , Chen J.-H., & Chen K.-H. (2025). The Molecular and Cellular Mechanisms of Endometriosis: From Basic Pathophysiology to Clinical Implications. *International Journal of Molecular Sciences*, 26, 2458. 10.3390/ijms26062458 40141102 PMC11941934

[xaag038-B18] Marquardt R. M. , Kim T. H., Shin J.-H., & Jeong J.-W. (2019). Progesterone and Estrogen Signaling in the Endometrium: What Goes Wrong in Endometriosis?. *International Journal of Molecular Sciences*, 20, 3822. 10.3390/ijms20153822 31387263 PMC6695957

[xaag038-B19] Neal C. L. , Yu D. (2010). 14-3-3zeta as a prognostic marker and therapeutic target for cancer. Expert Opinion on Therapeutic Targets, 14, 1343–1354. 10.1517/14728222.2010.531011 21058923 PMC3017465

[xaag038-B20] Pasalic E. , Tambuwala M. M., Hromic-Jahjefendic A. (2023). Endometriosis: Classification, pathophysiology, and treatment options. Pathol Res Pract, 251, 154847. 37844487 10.1016/j.prp.2023.154847

[xaag038-B21] Patel B. G. , Rudnicki M., Yu J., Shu Y., Taylor R. N. (2017). Progesterone resistance in endometriosis: origins, consequences and interventions. Acta Obstetricia et Gynecologica Scandinavica, 96, 623–632. 10.1111/aogs.13156 28423456

[xaag038-B22] Rahman M. S. , Hadrick K., Chung S. J., Carley I., Yoo J. Y., Nahar S., Kim T. H., Kim T., Jeong J. W. (2025a). Nanoceria as a non-steroidal anti-inflammatory drug for endometriosis theranostics. Journal of Controlled Release, 378, 1015–1029. 10.1016/j.jconrel.2024.12.074 39742921 PMC11830557

[xaag038-B23] Rahman M. S. , Kim T. H., Barrier B. F., Spencer T. E., Kelleher A. M., Jeong J. W. (2025b). FOXA2 loss results in an increase of endometriosis development and LIF reveals a therapeutic effect for endometriosis. FASEB J, 39, e70436. 40022603 10.1096/fj.202403182R PMC11926334

[xaag038-B24] Robinson M. D. , McCarthy D. J., Smyth G. K. (2010). edgeR: a Bioconductor package for differential expression analysis of digital gene expression data. Bioinformatics (Oxford, England), 26, 139–140. 10.1093/bioinformatics/btp616 19910308 PMC2796818

[xaag038-B25] Sigurgeirsson B. , Amark H., Jemt A., Ujvari D., Westgren M., Lundeberg J., Gidlof S. (2017). Comprehensive RNA sequencing of healthy human endometrium at two time points of the menstrual cycle. Biology of Reproduction, 96, 24–33. 10.1095/biolreprod.116.142547 28395321

[xaag038-B26] Teasley H. E. , Chang H. J., Kim T. H., Ku B. J., Jeong J. W. (2018). Expression of PIK3IP1 in the murine uterus during early pregnancy. Biochemical and Biophysical Research Communications, 495, 2553–2558. 10.1016/j.bbrc.2017.12.154 29289536 PMC5762254

[xaag038-B27] Teh W. T. , Chung J., Holdsworth-Carson S. J., Donoghue J. F., Healey M., Rees H. C., Bittinger S., Obers V., Sloggett C., Kendarsari R., Fung J. N., Mortlock S., Montgomery G. W., Girling J. E., Rogers P. A. W. (2023). A molecular staging model for accurately dating the endometrial biopsy. Nature Communications, 14, 6222. 10.1038/s41467-023-41979-z PMC10556104 37798294

[xaag038-B28] Zhang H. , Fang Y., Luo D., Li Y. H. (2025). Integration of single cell and bulk RNA-sequencing reveals key genes and immune cell infiltration to construct a predictive model and identify drug targets in endometriosis. Journal of Inflammation Research, 18, 2783–2804. 10.2147/JIR.S497643 40026309 PMC11871914

[xaag038-B29] Zhang P. , Wang G. (2023). Progesterone resistance in endometriosis: current evidence and putative mechanisms. International Journal of Molecular Sciences, 24 10.3390/ijms24086992 PMC10138736 37108154

[xaag038-B30] Zhao L. , Gu C., Ye M., Zhang Z., Han W., Fan W., Meng Y. (2017). Identification of global transcriptome abnormalities and potential biomarkers in eutopic endometria of women with endometriosis: A preliminary study. Biomedical Reports, 6, 654–662. 10.3892/br.2017.902 28584637 PMC5449958

[xaag038-B31] Zheng W. , Xiang D., Wen D., Luo M., Liang X., Cao L. (2023). Identification of key modules and candidate genes associated with endometriosis based on transcriptome data via bioinformatics analysis. Pathology, Research and Practice, 244, 154404. 10.1016/j.prp.2023.154404 36996608

