# Supplementary material for: YWHAZ loss is associated with endometrial dysfunction in proliferative-phase endometriosis
Source: Reproduction. 2026 Apr 6;171(4):xaag038. doi: 10.1093/reprod/xaag038 (PMC13058273; doi:10.1093/reprod/xaag038)
Supplement: xaag038_Supplementary_Data [file xaag038_supplementary_data.zip › Reproduction_Suppl Table 1 Human Endometriosis Proliferative Phase.pdf]

Supplemantary Table S1. Differentially expressed genes in eutopic endometrium of women with endometriosis during the proliferative phase.

Ensembl ID	Gene symbol	Description	Fold Change	FDR
ENST00000416762	EIF4B	eukaryotic translation initiation factor 4B	-173.45	0.04
ENST00000689239	CYB5R3	cytochrome b5 reductase 3	-136.12	0.04
ENST00000521309	YWHAZ	tyrosine 3-monooxygenase/tryptophan 5-monooxygenase activation protein zeta	-108.14	0.03
ENST00000518587	NPM1	Nucleophosmin 1	-90.09	0.00
ENST00000331272	FBXW4	F-box and WD repeat domain containing 4	-84.68	0.04
ENST00000679538	HSP90B1	heat shock protein 90 alpha family class B member 3	-65.87	0.00
ENST00000578157	MIR4657	microRNA 4657	-37.32	0.00
ENST00000481210	SEC61A1	SEC61 translocon subunit alpha 1	-21.10	0.01
ENST00000484488	PSMA7	proteasome 20S subunit alpha 7	-19.38	0.00
ENST00000552235	ZCRB1	zinc finger CCHC-type and RNA binding motif containing 1	-17.86	0.04
ENST00000453024	CTNNB1	catenin beta 1	-16.64	0.01
ENST00000482069	RPS24	ribosomal protein S24	-14.17	0.00
ENST00000677436	YKT6	YKT6 v-SNARE homolog	-13.17	0.03
ENST00000381298	IL6ST	interleukin 6 receptor	-10.60	0.00
ENST00000575069	P4HB	prolyl 4-hydroxylase subunit beta	-9.88	0.02
ENST00000432659	GSTO1	glutathione S-transferase omega 1	-9.19	0.04
ENST00000243040	PFDN5	prefoldin subunit 5	-8.36	0.00
ENST00000699275	SEC61A1	SEC61 translocon subunit alpha 1	-7.10	0.00
ENST00000439272	GNB1	G protein subunit beta 1	-6.73	0.02
ENST00000485947	VIM	vimentin	-5.48	0.01
ENST00000493477	FH	fumarate hydratase	-4.64	0.00
ENST00000531688	IFITM3	interferon induced transmembrane protein 3	-4.35	0.00
ENST00000396828	NEDD8	NEDD8 ubiquitin like modifier	-4.11	0.01
ENST00000463836	RPL22L1	ribosomal protein L22	-3.92	0.04
ENST00000295809	IFI16	interferon gamma inducible protein 16	-3.26	0.01
ENST00000511319	H2AZ1	H2A.Z variant histone 1	-2.97	0.04
ENST00000524349	RPS20	ribosomal protein S2	-2.51	0.00
ENST00000380381	RPS6	ribosomal protein S5	-2.49	0.03
ENST00000478340	RPL10A	ribosomal protein L10	-2.37	0.01
ENST00000521262	RPS20	ribosomal protein S20	-2.24	0.00
ENST00000481928	PTMA	prothymosin alpha	-2.17	0.01
ENST00000455785	STMN1	stathmin 1	2.09	0.00
ENST00000644185	RPL15	ribosomal protein L15	2.39	0.00
ENST00000574671	ACTG1	actin gamma 1	2.57	0.00
ENST00000585202	SRSF2	serine and arginine rich splicing factor 2	2.70	0.01
ENST00000533138	FTH1	ferritin heavy chain 1	4.88	0.00
ENST00000361521	ACOT7	acyl-CoA thioesterase 7	7.69	0.04
ENST00000585937	UQCR11	ubiquinol-cytochrome c reductase, complex III subunit XI	9.11	0.02
ENST00000460189	PGD	phosphogluconate dehydrogenase	9.31	0.04
ENST00000534719	FTH1	ferritin heavy chain 1	15.03	0.00
ENST00000522958	HNRNPH1	heterogeneous nuclear ribonucleoprotein F	32.85	0.00
ENST00000395699	PURB	pumilio RNA binding family member 3	73.07	0.00
ENST00000663894	TPM4	tropomyosin 4	142.28	0.00
ENST00000690835	CYB5R3	cytochrome b5 reductase 3	337.90	0.00
