# Supplementary material for: YWHAZ loss is associated with endometrial dysfunction in proliferative-phase endometriosis
Source: Reproduction. 2026 Apr 6;171(4):xaag038. doi: 10.1093/reprod/xaag038 (PMC13058273; doi:10.1093/reprod/xaag038)
Supplement: xaag038_Supplementary_Data [file xaag038_supplementary_data.zip › Supplemantary Figure 1.pdf]

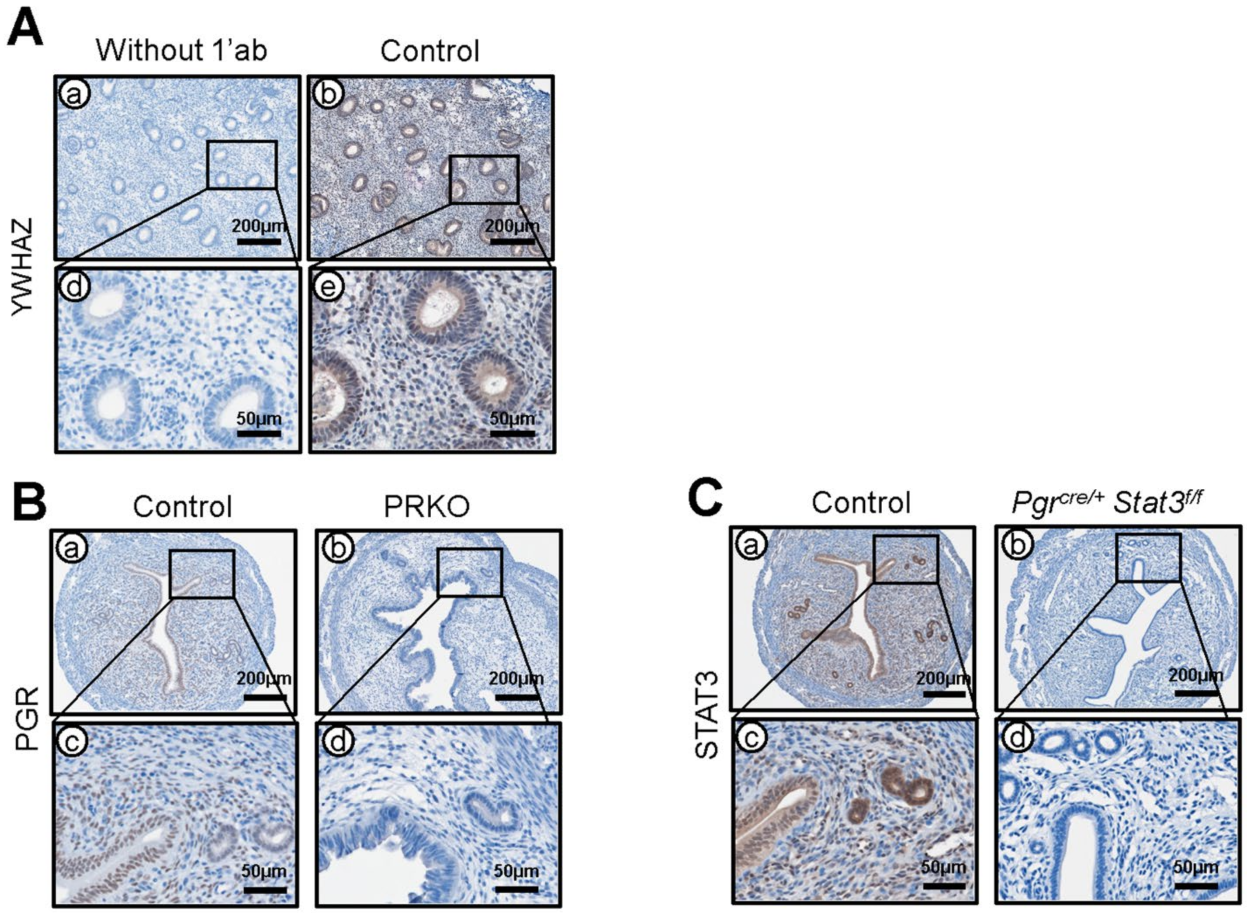

Supplementary Figure. 1 Immunohistochemical validation of YWHAZ antibody specificity and confirmation of PGR and STAT3 deletion in mouse uterine tissues. (A) Negative control for immunohistochemistry in proliferative phase human eutopic endometrium with/without YWHAZ primary antibody. (B) Immunohistochemical validation of Progesterone Receptor knockout (PRKO) mice showing absence of PGR in uterine tissue at gestational day (GD) 3.5. (C) Immunohistochemical confirmation of Signal Transducer and Activator of Transcription 3 (STAT3) loss in the uteri of *Pgr*$^{cre/+}$*Stat3*$^{f/f}$ mice at GD 3.5.
